# Healthcare providers’ use of dashboards with patient reported outcomes reinforces patients to fill out patient reported outcome measures

**DOI:** 10.1177/20552076241293975

**Published:** 2024-12-19

**Authors:** Annelieke Pasma, Céline van Lint, Monique Ardon-den Hollander, Sophie M. Bruinsma, Ingrid A. Peters

**Affiliations:** Department of Quality and Patient Care, Erasmus MC University Medical Center, Rotterdam, the Netherlands

**Keywords:** Patient-reported outcome measures, eHealth, health dashboard, consultation, treatment adherence

## Abstract

**Background:**

Patient reported outcomes are used to assess the impact of medical interventions on perceived health in both clinical trials and daily care. Further, patient-reported outcome measures (PROMs) optimize the consultation. However, patient completion of PROMs before their consultation, and healthcare provider's (HCP) use of PROMs in their consultation are suboptimal. In this cross-sectional study, we examined whether viewing a PROM dashboard before or during consultation consecutively resulted in higher patient completion of PROMs.

**Methods:**

As part of regular care, patients were asked to fill out PROMs prior their consultation. HCPs’ dashboard views were logged. A chi-square test was performed on dashboard viewed (yes/no) with consecutively filling out PROMs (yes/no). The odds ratio (OR) of consecutively filling out PROMs after a dashboard view was calculated.

**Results:**

38.016 consecutive appointments were linked to a previous appointment in which a dashboard could be viewed. In 2740 cases, a dashboard was viewed by the HCP, against 35.276 cases in which a dashboard was not viewed. Follow-up adherence in completing PROMs was 49%. The chi-square test showed statistical significance of *p* < .001. The OR of filling out a consecutive PROM when a dashboard was viewed was 3.16 (*p* < .001, 95% CI [2.9–3.5]).

**Discussion:**

Patients are more likely to complete PROMs for their follow-up appointment when an HCP has viewed PROMs during or before consultation. HCPs should be aware of their responsibility in addressing PROMs, because using PROMs in consultation does not only have a positive effect on communication, but also on patient completion of PROMs.

## Background

Patient-reported outcome measures (PROMs), assessing patients’ perceived health, are one of the important pillars on which the philosophy of value based healthcare is built.^1^ Value is defined as the health outcomes achieved per dollar spent.^1^ Next to their role in defining value in health, PROMs are becoming more and more widely used in clinical trials to assess the impact of medical interventions. In daily clinical care, this impact can also be measured with PROMs for the individual patient. They can be used to identify health issues and to follow-up on those issues. Despite the evident importance of assessing perceived health through the collection of PROMs, patient adherence in filling out PROMs can be lacking. As a result, the positive effects of PROMs are diminished, and bias in assessing value will be created. Up till now, the determinants of patient adherence in filling out PROMs are mostly unknown. Based on Feedback Intervention Theory, it is stated that when healthcare providers (HCPs) give patients feedback on their individual PROs, patients are more likely to subsequently fill out PROMs.^2,3^ With this study, we aim to provide empirical evidence for this relation.

Since the introduction of PROMs in the consultation room, the effect of PROMs on the medical consultation quality has been studied. The use of PROMs increased the identification of and discussion around health-related quality of life and resulted in patient–provider communication on more topics,^4,5^ especially on psychosocial and emotional domains.^4^ Overall, PROMs have a positive influence on patient–physician communication, and support dialog.^6,7^ Mixed results were found on the impact of using PROMs on quality of care.^4^ PROMs are an integrative and facilitative communication tool.^8^ They aid in assessing patient symptoms, facilitating clinical decision-making,^8^ and identifying symptoms outside the consultation.^6^

Effects of using PROMs on patients are less studied, but evidence suggests that PROMs feedback from the HCP improves a patients’ quality of life,^7^ diagnosis and notation,^7^ and disease control.^7^ Integration of PROMs in the consultation resulted in a trend toward higher patient satisfaction with care and communication.^5,9,10^ Not only patients, but HCPs were also more satisfied.^10–12^ Some HCPs may fear that discussing PROs during a medical consultation results in more consultation time. Research into the impact of PROMs on consultation time is undecisive,^12^ but does either show a reduction of consultation time^9^ or just no increase.^11^ Filling out PROMs can also have an effect on patients before the consultation takes place. PROM completion prompts patients to reflect on their health before their consultation as it encourages patients to raise issues with their HCP.^6^

While digitalization is becoming increasingly important in healthcare, PROMs are also digitalized into “electronic PROMs” (ePROMs). There are concerns that for patients with lower digital capabilities, ePROMs are not suitable. However, research showed that patient compliance in filling out ePROMs is equivalent to or even higher than for a standard paper PROM format^13,14^ Results on patient compliance in filling out ePROMs are varying. Filling out ePROMs with an app used by cancer patients resulted in 27.4% good compliance.^15^ A systematic review found a range between 61% and 96% with repetitive ePROMs, where the definition of adherence varied.^16^ Why patients do or do not fill out ePROMs is still a black box. It is believed that low literacy patients or patients from a non-Western cultural background are more likely to not adhere to ePROMs. When this is the case, it is even more important to improve patient adherence, since these patients often have lower health literacy, which is known to be related to disease outcomes.^17^ It is precisely for this patient population that discussing their health outcomes during their medical consultation can lead to higher health gains.

Literature shows a tendency of baseline adherence in filling out ePROMs to be higher than adherence in follow-up measurements.^18^ A lack of feedback on individual patient reported outcomes could play a role here. When patients do not receive feedback about their outcomes from HCPs, they do not find meaning in filling out ePROMs anymore. This phenomenon is called “identified regulation,” which occurs when a patient engages in an activity because he identifies with its value or meaning, in this case for their treatment, independent of the activity being pleasant or not.^19^ It suggests that receiving feedback about completed ePROMs, or at least knowing that the self-reported data was provided for a good reason, stimulates patients’ to provide this data for the following consultations.

Whether completing ePROMs is considered valuable seems at least partly dependent on the attitude of HCPs toward using ePROMs. Unfortunately, literature shows that HCPs experience several barriers in using ePROMs. Time constraints, insufficient staff, logistics, and financial resources are reported the most.^20^ Furthermore, technology, uncertainty about how or why to use ePROMs, and competing demands from established clinical workflows influence ePROM use by HCPs.^21^ HCPs might be unaware of their influence on patient adherence in filling out ePROMs.

A rich ePROM collection is not only beneficial for an individual patient, but a representative and inclusive sample of a patient population is also needed to measure patient value according to the value based health care theory, where patient value is defined by patient-relevant outcomes divided by costs per patient to achieve these outcomes.^1^ Therefore, it is essential to know which factors drive patient's adherence in filling out ePROMs. We hypothesize that there is a relation between HCPs paying attention to ePROMs during consultation, and patients’ filling out ePROMs for their consecutive medical appointment. The aim of this study is to examine the effect of HCPs viewing an ePROM dashboard on patients subsequently filling out ePROMs for their next appointment.

## Methods

This is a single site retrospective observational quality of care study in which logistic data on questionnaire adherence and dashboard views were analyzed. ePROM questionnaires and ePROM dashboards were introduced at the Erasmus MC University Medical Center outpatient clinics from July 2020 on.

### Data collection

The Erasmus MC University Medical Center outpatient clinics that implemented ePROMs in daily care, use the patient-reported outcomes measurement information system-10 (PROMIS-10) Global Health questionnaire for adults and the PROMIS Global Health 7 + 2 for children.^22^ Optionally, PROMIS short forms for depression, anxiety, fatigue, sleep disturbances, physical functioning, pain interference, and satisfaction with social roles and activities can be sent out to get a broader view on health. PROMIS short forms for cognitive functioning and peer relationships were sent out to parents of children between 5 and 18 years old, and, when aged between 8 and 18 years, to the children themselves too. The Computer Adaptive Testing (CAT) version of the “European Organization for Research and Treatment of Cancer QLQ-C30” (EORTC-QLQ-C30-CAT) was sent out to oncology patients.^23^ On top of these ePROMs, some departments send out other disease-specific ePROMs for their patients, for example, disease-specific quality of life questionnaires or questionnaires about specific symptoms. All licenses that were required to use the ePROMs in daily care were obtained from the copyright holders by the medical departments that are using these ePROMs. For disease-overarching questionnaires such as the PROMIS-10, the licenses were obtained by the department of Quality and Patient Care. An overview of ePROMs used in daily clinical care can be found in Supplemental File 1. In clinical care, mainly validated ePROMs are used, except when there is no validated ePROM available, for example, in case of rare diseases.

Nearly all patient communication at the Erasmus MC University Medical Center is digitalized. Patients need a two-step verification procedure for logging on to their secured online patient portal. Within this portal, appointment letters, laboratory results, information leaflets, and resumes of previous consultations can be viewed. Patients can also perform tasks, such as completing PROMs.

**Figure 1.**
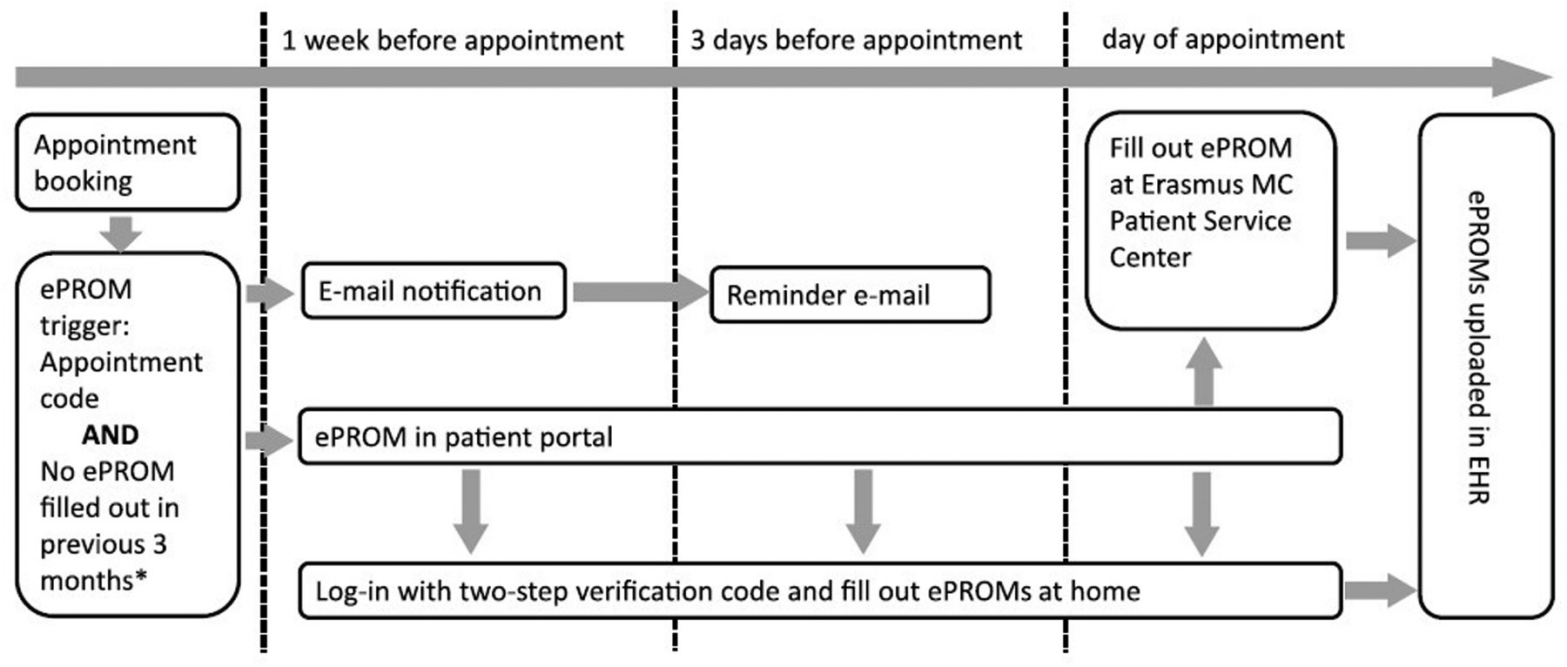
Automized logistics of sending out ePROMs to patients at the Erasmus MC University Medical Hospital Rotterdam.

*Note:* * 3 months is the standard timeframe in which ePROMs are sent out. Exceptions are made in accordance with HCPs. ePROMs: electronic patient-reported outcome measures; HCPs: healthcare providers.

Figure 1 visualizes the automized logistic process of sending out ePROMs to patients. Patients are requested through e-mail to log in to their secured patient environment and fill out ePROMs one week prior to their consultation. When patients have not completed all ePROMs before their consultation, a reminder e-mail is automatically sent out. For patients who do not have access to a computer or internet connection or have low digital skills, there is a Patient Service Center where patients can come to, to complete their ePROM(s), if needed with guidance from one of the volunteers.

**Figure 2.**
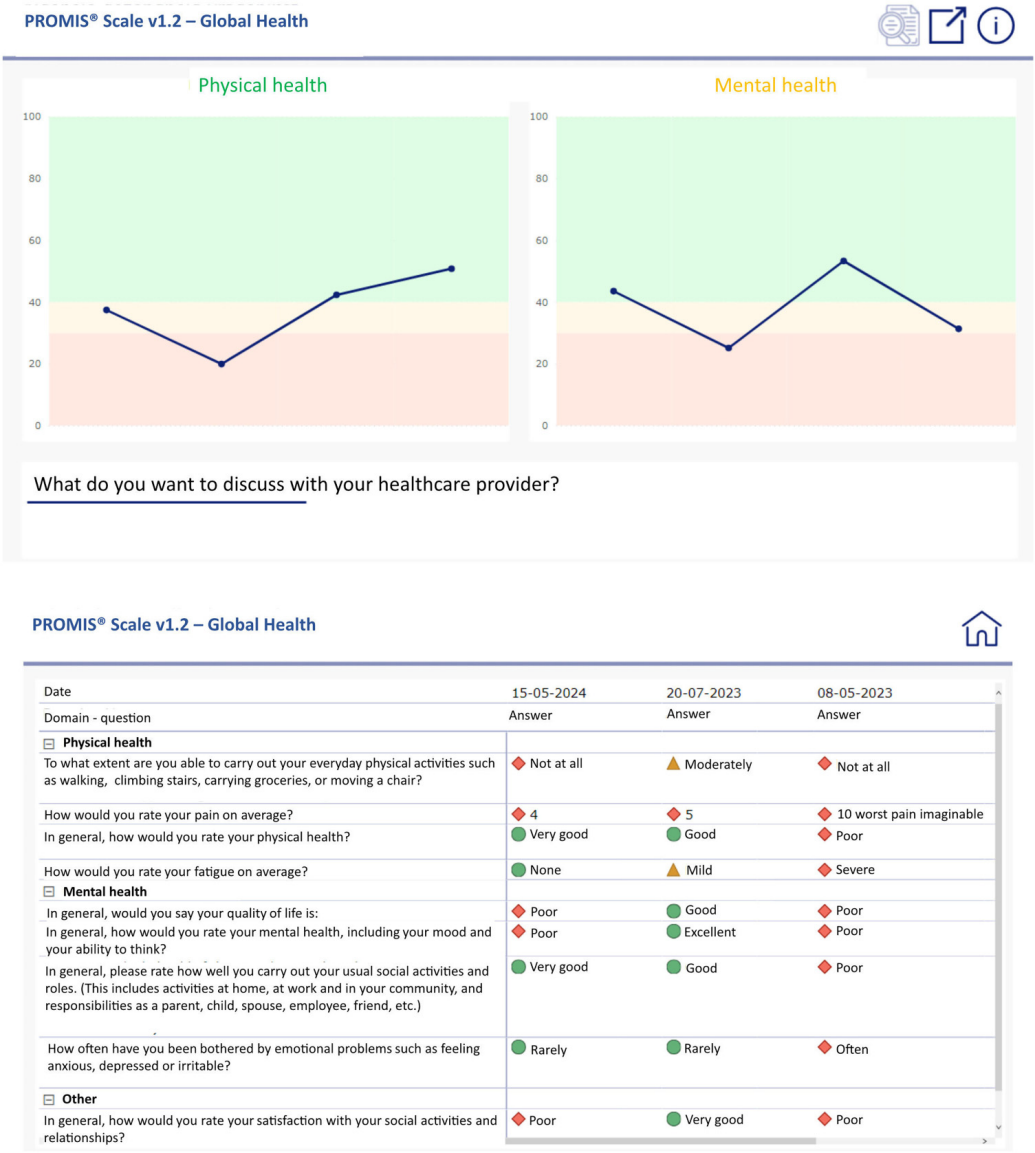
Dashboard visualization for PROMIS-10 questionnaire.

*Note:* Above: domain scores, below: scores on individual questions. PROMIS-10: patient-reported outcomes measurement information system.

All ePROMs are only sent out in case prespecified conditions are met, being a future appointment combined with a disease or treatment code. In addition, ePROMs are only sent out in case they had not been completed within the previous 3, 6, or 12 months (this time window is selected in accordance with the HCPs involved). Answers to ePROMs are stored in the electronic health record (EHR), accessible for HCPs only, and visualized in a dashboard through a viewer in the EHR. In this dashboard, sum scores of outcome domains, such as physical or mental health, are presented over time in a graph. The position of individual domain scores relative to norm scores is marked with colors (green, orange, red). HCPs can zoom in on the answers underlying the domain scores by clicking on the “zoom”-icon. In Figure 2, an example of a generic PROMIS-10 dashboard is given.

All HCPs that have access to PROM dashboards are given verbal and written instructions about using them. Group training about using PROMs for shared decision making, coaching on the job, and feedback on their performance in using dashboards is available on demand.

When an HCP accesses a PROM dashboard, this is logged in an SQL-table, in a data platform where all data from the EHR is stored. We collected logging data from dashboard views from October 2022 until December 2023. These data show when and for which patient a PROM dashboard was viewed and by whom. HCP and patient identifying data was anonymized. For the same time period, patients’ adherence data of completing ePROMs was logged together with the scheduled date of their appointment. All ePROM adherence data was matched with dashboard logging data. A match was defined as a dashboard view within one week of the appointment date. We used this one-week time interval, since HCPs also view PRO dashboards in preparation of the consultation. The ePROM data collection was submitted to the Erasmus MC local medical ethics board. Since the data collection is being used primarily for clinical care, the Dutch law on medical scientific research does not apply. Therefore, the Erasmus MC medical ethics board provided a waiver (MEC-2020-0039). Written informed consent from patients was not needed, because this is a retrospective investigation into routine logistic healthcare processes and all data was processed anonymously.

### Data analysis

All patients that were offered an ePROM before their medical consultation were anonymously included in the analysis. For the analysis in which the odds ratio (OR) was calculated, all patients that filled out an ePROM questionnaire at least once and were offered a second ePROM questionnaire for a consecutive follow-up appointment were anonymously included. All HCPs that viewed a dashboard were anonymously included in the analysis.

**Figure 3.**
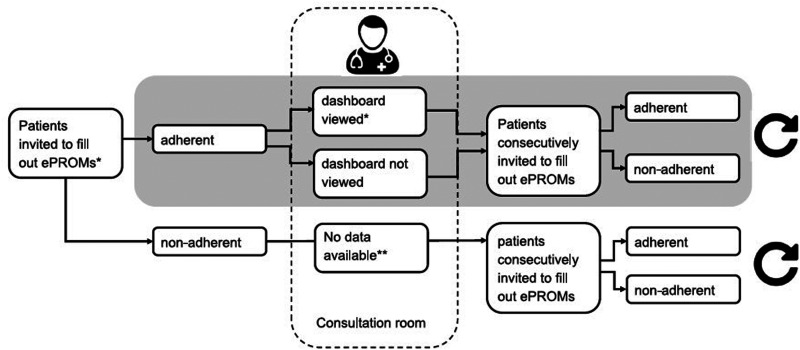
Flow chart of data included in the analysis.

*Note:* Gray area: sample used for chi-square test and odds ratio calculation. *: viewed during consultation or in preparation of the consultation; **: when ePROMs are not completed, there is no dashboard data available. Abbreviation: ePROMs: electronic patient-reported outcome measures.

No patients or HCPs were excluded from the analysis. The data collection procedure is visualized in Figure 3.

Since this is a retrospective analysis of healthcare data and covers a large amount of data, a confirmatory sample size calculation was performed. For the chi-square test, with an expected three times higher probability of filling out consecutive ePROMs in the group where dashboards were viewed, a sample of at least 245 patients per group is needed, taking into account an alpha of 0.05 and a power of 0.8. For the OR calculation, with a relative precision of 50%, 95% CI, an expected prevalence of viewing dashboards of 5%, expected odds of 3, and a case ratio of 1 to 12, a sample of at least 2088 patients is needed. Duplicate dashboard logs (e.g. when an HCP accessed the same dashboard twice) were removed. Data are presented as count and percentage for categorical data and mean and standard deviation for continuous, approximately normally distributed data.

To assess differences in consecutive patient adherence in completing ePROMs between the group of HCPs that viewed a dashboard and did not view a dashboard, we constructed a two-by-two table in which logging data (dashboard viewed yes/no) and consecutive questionnaire filled out by the patient (yes/no) were combined. A chi-square test was performed on this data.

To evaluate the effect of an HCP viewing the dashboard on consecutively completing an ePROM by the patient, a binomial regression analysis with stepwise backward selection was performed with dashboard viewed as dummy variable, age and sex, and consecutively completing an ePROM as dependent variable.

All analyses were performed using IBM SPSS version 28.01.

## Results

**Figure 4.**
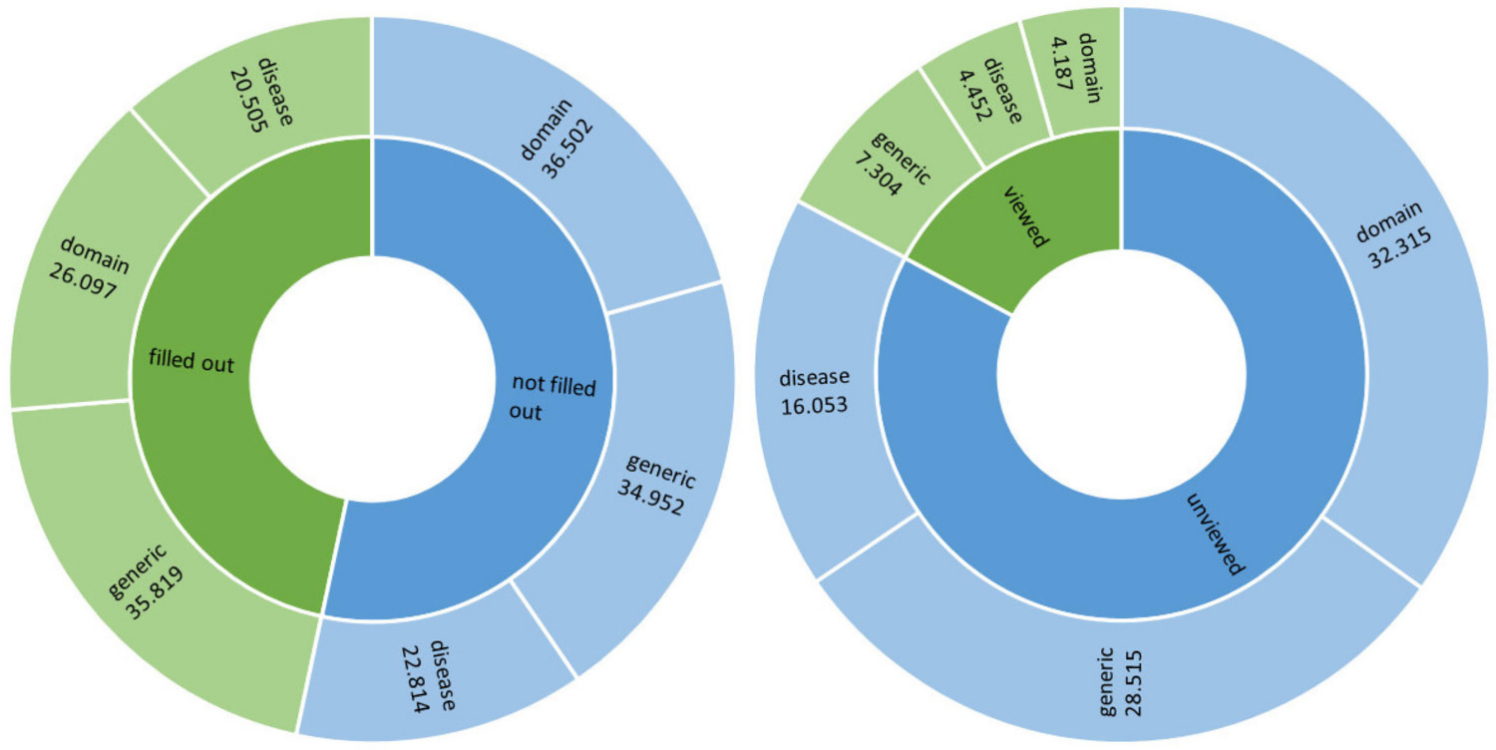
ePROM adherence (*n*) (left) and dashboard views (*n*) (right) per ePROM type (generic ePROMs, domain-specific ePROMs, disease-specific ePROMs) from October 2022 to December 2023.

Abbreviation: ePROMs: electronic patient-reported outcome measures.

From October 2022 to December 2023, a total of 42.976 unique patients were identified (male: 46.9%, mean age: 50.44) (see Table 1). A total of 176,800 questionnaires were sent out, consisting of 40% generic ePROMs, 35.5% domain-specific ePROMs and 24.5% disease-specific ePROMs. Overall patient adherence in completing ePROMs was 46.7%. For generic, domain-specific and disease-specific ePROMs adherence was respectively 50.6%, 41.7%, and 47.3%. The degree of adherence in completing disease-specific ePROMs varied, ranging from 20% (oral surgery) to 71.3% (Turner syndrome). In Figure 4, adherence in completing ePROMs is visualized and divided over the three ePROM levels (generic, domain-specific, disease-specific).

**Table 1.** Patient baseline characteristics, ePROM characteristics and dashboard views.

No of unique patients	42,976
Age, mean (SD)	50.44 (22.3)
Gender, male, %	46.9
No of appointments with ePROMs received, *n* (%)	
1	25,000 (58.2)
2	9,400 (21.9)
3	4,139 (9.6)
4	1996 (4.6)
5	986 (2.3)
>5 times	1,455 (3.4)
Amount of ePROMs received per timepoint, *n* (%)	
1	22,613 (27.7)
2	33,689 (41.3)
≥3*	25,357 (31)
No of dashboard views per consultation, *n* (%)	
1	5966 (58.8)
2	2722 (26.8)
≥3	1466 (14.4)

Abbreviations: ePROMs: electronic patient-reported outcome measures; SD: standard deviation.

*Including parent proxy forms.

On questionnaire level, 19.3% of all completed ePROMs were viewed in a dashboard. 20.4% of all completed generic ePROMs were viewed in a dashboard, 16% of domain-specific completed ePROMs and 21.7% of disease-specific completed ePROMs. Figure 4 visualizes the dashboard views of completed ePROM questionnaires, divided over three levels (generic, domain-specific, disease-specific).

For 6722 of 42,976 unique patients (15.6%), at least one dashboard had been viewed in the week before or on the day of their outpatient appointment. In 6633 of 80,992 outpatient appointments (8.2%) at least one dashboard had been viewed. Note that one patient may have had several appointments during our measurement period.

A total of 38,016 consecutive appointments could be linked to a previous appointment in which a dashboard could have been viewed (i.e. the patient had completed at least one questionnaire). In 2740 cases, a dashboard had been viewed during or in the week before the consultation, against 35,276 cases where no dashboard had been viewed. In 4768 consecutive appointments, one or more questionnaires had been filled out (49%). A chi-square test (see Table 2) had a statistical significance of *p* < .001.

**Table 2.** 2 × 2 table on dashboard viewed by healthcare provider (yes/no), and consecutively completing at least a PROM by patients.

	Consecutive completion of ePROM by patient, *n* (%)			
		No	Yes	Total
Viewing a dashboard by HCP	No	18.682 (49.1)	16.594 (43.7)	35.276 (92.8)
	Yes	720 (1.9)	2020 (5.3)	2740 (7.2)
	Total	19.402 (51)	18.614 (49)	38.016 (100)
Chi-square test: *p* < .001.				

Abbreviations: HCP: healthcare provider, PROM: patient reported outcome measure; ePROMs: electronic PROMs.

A binomial regression with stepwise backward selection was run with independent variables “dashboard viewed” (yes/no), age and sex and consecutive completion of ePROMs as dependent variable. Age and sex were not found statistically significant predictors and were retracted from the model. The final model with the variable “dashboard viewed” resulted in an OR of 3.16 (*p* < .001, 95% CI [2.9–3.5]), showing that the chance of completing an ePROM in preparation of a follow-up appointment was more than three times higher in case the HCP had checked ePROM results in the dashboards during or in preparation of a previous consultation.

## Discussion

Patients are more likely to complete ePROMs when their HCP pays attention to PROs during consultation. To the best of our knowledge, this is the first study that contributes to empirical evidence on the effect of paying attention to patient reported outcomes during consultation on patients’ willingness to complete consecutive ePROMs.

We investigated whether HCPs viewed ePROMs that were completed by patients. Our results show that patients are three times more likely to fill out consecutive ePROMs when their HCP has viewed the dashboard during or in preparation of their previous consultation. This finding supports the theory that receiving feedback on a task makes people more likely to fulfill a follow up task.^2,3^ Patients find meaning in filling out ePROMs when they are paid attention to by their HCP.

However, feedback can come in many ways. If patients are mainly interested in their own outcomes, having access to a dashboard showing their results themselves could be sufficient. This was not the case during the time period in which the current data was collected. Patients were dependent on their HCP for insight into their results. But previous research shows that even when patients have direct access to their ePROM outcomes, they might need a healthcare professional for guidance as they can experience difficulty with understanding the way in which outcomes are represented (e.g. graphs).^24^ It could be that the HCP has a key mediating role in discussing PROM results, giving meaning to and interpreting these outcomes.

Based on our main finding, it could also be that when HCPs pay attention to a patient's nonadherence in completing ePROMs, this might positively impact a patient's subsequent adherence. Targeted interventions are needed to specifically address determinants of not completing ePROMs. Determinants may be not knowing how to fill out ePROMs, or lacking knowledge on why one is requested to complete ePROMs in the first place. Interestingly, there is still a group of patients who keeps on filling out ePROMs, even when they were not used in their consultation by their HCP. Previous research^6^ suggests that for these patients ePROMs could serve as a guided preparation for their consultation. Patients might become aware of how they are actually feeling and may prepare questions for their medical specialist.

The percentage of PROM dashboards that were viewed by HCPs is too low to consider ePROMs being sufficiently implemented in the Erasmus MC consultation rooms. Following Rogers’ innovation adoption curve, implementation takes a rise when the early majority has adopted the innovation.^25^ At the time of writing, adoption of using PROM dashboards in daily care is at the level of early adopters. To take the early majority along, the determinants of discussing ePROMs by HCPs need to be studied further. This way, targeted interventions can be set up. Patients’ compliance level may play a role here. As compliance was on average less than 50%, HCPs may have become demotivated to view PROM dashboards, after having encountered several patients with uncompleted ePROMs. Other determinants could be the absence of active alerts of completed ePROMs in the EHR, resulting in unawareness about the possibility to view PROM dashboards.

As ePROM questionnaires and PROM dashboards have been introduced at the Erasmus MC University Medical Center from July 2020 on, we have supported many different medical departments during implementation since. Our experience shows that some medical departments integrate the use of PROM dashboards in consultation practice easier than others. This might be due to several factors. First, the design of the care pathway might influence the use of ePROMs. For example, when there is an additional consultation with a specialized nurse, there is more time available to discuss PROM outcomes. Second, for some medical conditions, the use of ePROMs is more common and needed for medical decision making than for others. Thus, when implementing ePROMs, there should be awareness about how ePROMs fit in the care pathway (e.g. who is going to discuss the outcomes?) and which patient reported outcomes add relevant information to and are needed for medical decision making.

HCP's knowledge on how to handle PROMs during their consultations is a prerequisite for sufficient implementation of PROMs in their conversations with patients.^26^ However, research on how to integrate PROMs in a consultation, for example in guiding shared decision making, agenda-setting and aiding a patient-centered consultation, is lacking. We suggest that HCP training in how to address PROMs during consultation can be beneficial, as is peer-to-peer communication about how to handle PROMs in patient care and the benefits of discussing outcomes with patients. Interventions should be targeted at the determinants of not performing the desirable behavior, being discussing PROMs during consultation. Proven methods for studying determinants and developing accompanied interventions, such as Intervention Mapping, may be used.^27^

Although the OR of 3.16 indicates a robust result, there are some limitations that need to be considered. We used logging data of HCPs’ dashboard views as a proxy for discussing PROMs during consultation. However, we cannot be certain that PROMs were actually discussed in case a dashboard had been viewed. For example, it could be that PROMs were monitored in preparation of the consultation, but were not actually paid attention to during consultation. As we could not make this distinction, the real effect of paying attention to PROM outcomes on consecutive ePROM adherence might even be larger. Furthermore, even when an HCP paid attention to PROMs, we do not know whether this was of added value to the patient–HCP conversation.

To validate the use of dashboard views as a proxy for paying attention to outcomes during a consultation, research should focus on the observation of PROM use in the consultation room. Consultations can be audiotaped and scored using validated observation instruments, for example for shared decision-making, such as the OPTION-5.^28^ Unfortunately, up till now there are no validated observation instruments available to score the use of PROMs and their added value in shared decision making or patient-centered care during consultations.

Despite these uncertainties, data logs give us the opportunity to explore relationships within a large number of observations, which would otherwise have remained undetermined.

## Conclusion

This study shows that patients are 3.16 times more likely to complete ePROMs when HCPs had viewed a PROM dashboard during or in preparation of their previous consultation. HCPs should be aware of their role and responsibility in addressing PROMs during consultation, because using PROMs does not only have a positive effect on communication, as has been shown by previous studies,^4–10^ but also on patient adherence in filling out ePROMs.

## Supplemental Material

sj-docx-1-dhj-10.1177_20552076241293975 - Supplemental material for Healthcare providers’ use of dashboards with patient reported outcomes reinforces patients to fill out patient reported outcome measures Supplemental material, sj-docx-1-dhj-10.1177_20552076241293975 for Healthcare providers’ use of dashboards with patient reported outcomes reinforces patients to fill out patient reported outcome measures by Annelieke Pasma, Céline van Lint, Monique Ardon-den Hollander, Sophie M. Bruinsma and Ingrid A. Peters in DIGITAL HEALTH
