# Supplementary material for: Healthcare providers’ use of dashboards with patient reported outcomes reinforces patients to fill out patient reported outcome measures
Source: Digit Health. 2024 Dec 19;10:20552076241293975. doi: 10.1177/20552076241293975 (PMC12032432; doi:10.1177/20552076241293975)
Supplement: sj-docx-1-dhj-10.1177_20552076241293975 - Supplemental material for Healthcare providers’ use of dashboards with patient reported outcomes reinforces patients to fill out patient reported outcome measures [file sj-docx-1-dhj-10.1177_20552076241293975.docx]

***Supplementary file 1.***

***Listing of ePROMs used in daily care at the Erasmus MC University Medical Center Rotterdam, the Netherlands***

- Basal and Squamous Cell Carcinoma Quality of Life questionnaire (BaSQoL) ^1^
- Breast-Q^2^
- Child Health Questionnaire 45 (CHQ)^3^
- Common Variable Immunodeficiency Questionnaire Quality of Life Questionnaire (CVID QoL)^4^
- Disease-Specific Health-Related Quality of Life Questionnaire (DTF-QoL) for Patients with Desmoid-Type Fibromatosis^5^
- Endometriosis Health Profile long-form 30 (EHP-30)^6^
- European Organization for Research and Treatment of Cancer – breast cancer-specific quality-of-life questionnaire module (EORTC-BR23)^7^
- FertiQoL^8^
- European Organization for Research and Treatment of Cancer - muscle invasive bladder cancer quality-of-life questionnaire module (EORTC QLQ BLM 30)^9^
- European Organization for Research and Treatment of Cancer - Brain cancer quality-of-life questionnaire module (EORTC QLQ-BN20)^10^
- European Organization for Research and Treatment of Cancer - EORTC QLQ-C30 CAT^11^
- European Organization for Research and Treatment of Cancer - hepatocellular carcinoma-specific quality of life questionnaire module (EORTC QLQ-HCC18)^12^
- European Organization for Research and Treatment of Cancer – lung cancer quality-of-life questionnaire module (EORTC QLQ-LC13)^13^
- European Organization for Research and Treatment of Cancer - health-related quality of life questionnaire module in patients with cancer of the oesophagus, the oesophago-gastric junction and the stomachEORTC (QLQ-OG25)^14^
- European Organization for Research and Treatment of Cancer - health-related quality of life questionnaire module in patients with gastrointestinal neuroendocrine tumours (EORTC QLQ – GI.NET21)^15^
- Early-Onset Scoliosis-24-Item Questionnaire (EOSQ-24)^16^
- EQ-5D-3L-Y^17^
- EQ-5D-3L-Y - Proxy^18^
- Functional Assessment of Cancer Therapy - Endocrine Symptoms (FACT-ES)^19^
- Self-Administered Comorbidity Questionnaire (SACQ) ^20^
- Quality of Life Questionnaire-Bronchiectasis (QOL-B)^21^
- revised Cystic Fibrosis Questionnaire (CFQ-R)^22^
- ITQOL-SF47^23^
- Dutch Facial Clinimetric Evaluation Scale (FaCE Scale)^24^
- Synkinesis Assessment Questionnaire (SAQ)^25^
- WoundQoL^26^
- VEINES-QOL^27^
- Frailty Groningen Indicator^28^
- Geriatric 8 (G8)^29^
- Hospital Anxiety and Depression Scale (HADS)^30^
- Neuro-QoL item bank – Stigma^31^
- National Survey of Sexual Attitudes and Lifestyles (NATSAL)^32^
- Infant and Toddler Quality of Life Questionnaire Short Form 47 (ITQOL-SF47)^23^
- Ready-Steady-Go-Hello^33^
- Transition Readiness Questionnaire (TRAQ)^34^
- Vancouver Symptom Score^35^
- Quality of life measure for children with bladder dysfunction (PinQ)^36^
- Utrecht Questionnaire for Outcome Assessment in Aesthetic Rhinoplasty (D-OAR)^37^
- FACE-Q Rhinoplasty module^38^
- Standardized Cosmesis and Health Nasal Outcomes Survey (SCHNOS)^39^
- Distress Thermometer^40^
- Mastocytosis Symptom Assessment Form (MSAF)^41^
- Memorial Anxiety Scale for Prostate Cancer (MAX PC)^42^
- European Organisation for Research and Treatment of Cancer – Quality of Life Questionnaire module for patients with Multiple Myeloma (EORTC- QLQ- MY-20)^43^
- Patient-Reported Outcomes version of the Common Terminology Criteria for Adverse Events (NCI-PRO-CTCAE)^44^
- Oral Health Impact Profile (OHIP-14)^45^
- PAID-5^46^
- Pediatric Quality of Life Inventory (PedsQL)^47^
- Work Ability Index (WAI)^48^
- Patient-Reported Outcomes Measurement Information System Short Form Emotional Support (PROMIS-ES)^49^
- Greene Climacteric Scale (GCS)^50^
- MARS-5^51^
- Beck Depression Inventory^52^
- Patient-Reported Outcomes Measurement Information System Scale v1.2: global health (PROMIS-10)^53^
- Patient-Reported Outcomes Measurement Information System Pediatric Global Health scale (PGH-7)^54^
- Patient-Reported Outcomes Measurement Information System Scale Fatigue Short Form^49^
- Patient-Reported Outcomes Measurement Information System Scale Depression Short Form^49^
- Patient-Reported Outcomes Measurement Information System Scale Anxiety Short Form^49^
- Patient-Reported Outcomes Measurement Information System Scale Sleep Disturbances Short Form^49^
- Patient-Reported Outcomes Measurement Information System Scale Physical Functioning Short Form^49^
- Patient-Reported Outcomes Measurement Information System Scale Pain Interference Short Form^49^
- Patient-Reported Outcomes Measurement Information System Scale Satisfaction with Social Roles and Activities Short Form^49^
- Patient-Reported Outcomes Measurement Information System Scale Pediatric Cognitive Functioning^55^
- Patient-Reported Outcomes Measurement Information System Scale Pediatric Peer relationships^55^
- Patient-Reported Outcomes Measurement Information System Scale Pediatric Anxiety^56^
- Patient-Reported Outcomes Measurement Information System Scale Pediatric Depressive symptoms^56^
- Patient-Reported Outcomes Measurement Information System Scale Pediatric Physical Function - Upper Extremity Function^55^
- Spine Oncology Study Group Outcomes Questionnaire (SOSGOQ)^57^
- Scoliosis Research Society 22-revised (SRS-22r)^58^
- The Older Persons and Informal Caregivers Survey Short Form (TOPICS-SF)^59^
- Basel Assessment of Adherence to Immunosuppressive Medications Scale (BAASIS)^60^
- International Prostate Symptom Score (IPSS)^61^
- Visual Functioning Questionnaire 39 (VFQ-39)^62^
- Edinburgh Postnatal (Postpartum) Depression Scale (EPDS)^63^
- International Consultation on Incontinence Questionnaire – Urinary Incontinence Short Form (ICIQ-UI-SF)^64^
- Wexner (Cleveland Clinic) Score^65^
- Birth Satisfaction Scale-Revised (BSS-R)^66^
- Mother Infant Bonding Scale (MIBS)^67^
- The Breastfeeding Self-Efficacy Scale-Short Form (BSES-SF)^68^

***References***

1. Waalboer-Spuij R, Hollestein LM, Timman R, et al. Development and validation of the basal and squamous cell carcinoma quality of life (BaSQol) questionnaire. *Acta Dermato-Venereologica* 2018; 98: 234-239. Article. DOI: 10.2340/00015555-2806.

2. Pusic AL, Klassen AF, Scott AM, et al. Development of a new patient-reported outcome measure for breast surgery: The BREAST-Q. *Plastic and Reconstructive Surgery* 2009; 124: 345-353. Article. DOI: 10.1097/PRS.0b013e3181aee807.

3. Wulffraat N, Van Der Net JJ, Ruperto N, et al. The Dutch version of the Childhood Health Assessment Questionnaire (CHAQ) and the Child Health Questionnaire (CHQ). *Clinical and Experimental Rheumatology* 2001; 19: S111-S115. Article.

4. Quinti I, Pulvirenti F, Giannantoni P, et al. Development and Initial Validation of a Questionnaire to Measure Health-Related Quality of Life of Adults with Common Variable Immune Deficiency: The CVID_QoL Questionnaire. *Journal of Allergy and Clinical Immunology: In Practice* 2016; 4: 1169-1179.e1164. Article. DOI: 10.1016/j.jaip.2016.07.012.

5. Schut ARW, Lidington E, Timbergen MJM, et al. Development of a Disease-Specific Health-Related Quality of Life Questionnaire (DTF-QoL) for Patients with Desmoid-Type Fibromatosis. *Cancers* 2022; 14. Article. DOI: 10.3390/cancers14030709.

6. Van De Burgt TJM, Hendriks JCM and Kluivers KB. Quality of life in endometriosis: Evaluation of the Dutch-version Endometriosis Health Profile-30 (EHP-30). *Fertility and Sterility* 2011; 95: 1863-1865. Article. DOI: 10.1016/j.fertnstert.2010.11.009.

7. Sprangers MA, Groenvold M, Arraras JI, et al. The European Organization for Research and Treatment of Cancer breast cancer-specific quality-of-life questionnaire module: First results from a three-country field study. *Journal of Clinical Oncology* 1996; 14: 2756-2768. Article. DOI: 10.1200/jco.1996.14.10.2756.

8. Aarts JWM, Van Empel IWH, Boivin J, et al. Relationship between quality of life and distress in infertility: A validation study of the Dutch FertiQoL. *Human Reproduction* 2011; 26: 1112-1118. Article. DOI: 10.1093/humrep/der051.

9. Ripping TM, Rammant E, Witjes JA, et al. Validation and reliability of the Dutch version of the EORTC QLQ-BLM30 module for assessing the health-related quality of life of patients with muscle invasive bladder cancer. *Health and Quality of Life Outcomes* 2022; 20. Article. DOI: 10.1186/s12955-022-02064-z.

10. Taphoorn MJB, Claassens L, Aaronson NK, et al. An international validation study of the EORTC brain cancer module (EORTC QLQ-BN20) for assessing health-related quality of life and symptoms in brain cancer patients. *European Journal of Cancer* 2010; 46: 1033-1040. Article. DOI: 10.1016/j.ejca.2010.01.012.

11. Petersen MA, Aaronson NK, Arraras JI, et al. The EORTC CAT Core—The computer adaptive version of the EORTC QLQ-C30 questionnaire. *European Journal of Cancer* 2018; 100: 8-16. Article. DOI: 10.1016/j.ejca.2018.04.016.

12. Chie WC, Blazeby JM, Hsiao CF, et al. International cross-cultural field validation of an European Organization for Research and Treatment of Cancer questionnaire module for patients with primary liver cancer, the European Organization for Research and Treatment of Cancer quality-of-life questionnaire HCC18. *Hepatology* 2012; 55: 1122-1129. Article. DOI: 10.1002/hep.24798.

13. Bergman B, Aaronson NK, Ahmedzai S, et al. The EORTC QLQ-LC13: a modular supplement to the EORTC core quality of life questionnaire (QLQ-C30) for use in lung cancer clinical trials. *European Journal of Cancer* 1994; 30: 635-642. Article. DOI: 10.1016/0959-8049(94)90535-5.

14. Lagergren P, Fayers P, Conroy T, et al. Clinical and psychometric validation of a questionnaire module, the EORTC QLQ-OG25, to assess health-related quality of life in patients with cancer of the oesophagus, the oesophago-gastric junction and the stomach. *European Journal of Cancer* 2007; 43: 2066-2073. Article. DOI: 10.1016/j.ejca.2007.07.005.

15. Yadegarfar G, Friend L, Jones L, et al. Validation of the EORTC QLQ-GINET21 questionnaire for assessing quality of life of patients with gastrointestinal neuroendocrine tumours. *British Journal of Cancer* 2013; 108: 301-310. Article. DOI: 10.1038/bjc.2012.560.

16. Wijdicks SPJ, Dompeling SD, De Reuver S, et al. Reliability and Validity of the Adapted Dutch Version of the Early-Onset Scoliosis-24-Item Questionnaire (EOSQ-24). *Spine* 2019; 44: E965-E973. Article. DOI: 10.1097/brs.0000000000003017.

17. Ramos-Goñi JM, Oppe M, Stolk E, et al. International Valuation Protocol for the EQ-5D-Y-3L. *PharmacoEconomics* 2020; 38: 653-663. Article. DOI: 10.1007/s40273-020-00909-3.

18. Fitriana TS, Purba FD, Stolk E, et al. EQ-5D-Y-3L and EQ-5D-Y-5L proxy report: psychometric performance and agreement with self-report. *Health and Quality of Life Outcomes* 2022; 20. Article. DOI: 10.1186/s12955-022-01996-w.

19. Fallowfield LJ, Leaity SK, Howell A, et al. Assessment of quality of life in women undergoing hormonal therapy for breast cancer: Validation of an endocrine symptom subscale for the FACT-B. *Breast Cancer Research and Treatment* 1999; 55: 189-199. Article. DOI: 10.1023/a:1006263818115.

20. Sangha O, Stucki G, Liang MH, et al. The Self-Administered Comorbidity Questionnaire: A new method to assess comorbidity for clinical and health services research. *Arthritis Care and Research* 2003; 49: 156-163. Article. DOI: 10.1002/art.10993.

21. Quittner AL, O'Donnell AE, Salathe MA, et al. Quality of Life Questionnaire-Bronchiectasis: Final psychometric analyses and determination of minimal important difference scores. *Thorax* 2015; 70: 12-20. Article. DOI: 10.1136/thoraxjnl-2014-205918.

22. Quittner AL, Sawicki GS, McMullen A, et al. Psychometric evaluation of the cystic fibrosis questionnaire-revised in a national sample. *Quality of Life Research* 2012; 21: 1267-1278. Article. DOI: 10.1007/s11136-011-0036-z.

23. Landgraf JM, Vogel I, Oostenbrink R, et al. Parent-reported health outcomes in infants/toddlers: Measurement properties and clinical validity of the ITQOL-SF47. *Quality of Life Research* 2013; 22: 635-646. Article. DOI: 10.1007/s11136-012-0177-8.

24. Kleiss IJ, Beurskens CHG, Stalmeier PFM, et al. Quality of life assessment in facial palsy: validation of the Dutch Facial Clinimetric Evaluation Scale. *European Archives of Oto-Rhino-Laryngology* 2015; 272: 2055-2061. Article. DOI: 10.1007/s00405-015-3508-x.

25. Kleiss IJ, Beurskens CHG, Stalmeier PFM, et al. Synkinesis assessment in facial palsy: validation of the Dutch Synkinesis Assessment Questionnaire. *Acta Neurologica Belgica* 2016; 116: 171-178. Article. DOI: 10.1007/s13760-015-0528-7.

26. Amesz SF, Klein TM, Meulendijks AM, et al. A translation and preliminary validation of the Dutch Wound-QoL questionnaire. *BMC Dermatology* 2020; 20. Article. DOI: 10.1186/s12895-020-00101-2.

27. van der Velden SK, Biemans AAM, Nijsten T, et al. Translation and validation of the Dutch VEINES-QOL/Sym in varicose vein patients. *Phlebology* 2014; 29: 227-235. Article. DOI: 10.1177/0268355513476279.

28. Metzelthin SF, Danils R, Van Rossum E, et al. The psychometric properties of three self-report screening instruments for identifying frail older people in the community. *BMC Public Health* 2010; 10. Article. DOI: 10.1186/1471-2458-10-176.

29. Bellera CA, Rainfray M, Mathoulin-Pélissier S, et al. Screening older cancer patients: First evaluation of the G-8 geriatric screening tool. *Annals of Oncology* 2012; 23: 2166-2172. Article. DOI: 10.1093/annonc/mdr587.

30. Spinhoven P, Ormel J, Sloekers PPA, et al. A validation study of the hospital anxiety and depression scale (HADS) in different groups of Dutch subjects. *Psychological Medicine* 1997; 27: 363-370. Article. DOI: 10.1017/s0033291796004382.

31. Cella D, Lai JS, Nowinski CJ, et al. Neuro-QOL: Brief measures of health-related quality of life for clinical research in neurology. *Neurology* 2012; 78: 1860-1867. Article. DOI: 10.1212/WNL.0b013e318258f744.

32. Mitchell KR, Palmer MJ, Lewis R, et al. Development and Validation of a Brief Measure of Sexual Wellbeing for Population Surveys: The Natsal Sexual Wellbeing Measure (Natsal-SW). *Journal of Sex Research* 2023. Article. DOI: 10.1080/00224499.2023.2278530.

33. Nagra A, McGinnity PM, Davis N, et al. Implementing transition: Ready Steady Go. *Archives of Disease in Childhood: Education and Practice Edition* 2015; 100: 313-320. Article. DOI: 10.1136/archdischild-2014-307423.

34. van Gaalen MAC, van Gijn E, van Pieterson M, et al. Validation and Reference Scores of the Transition Readiness Assessment Questionnaire in Adolescent and Young Adult IBD Patients. *Journal of pediatric gastroenterology and nutrition* 2023; 77: 381-388. Article. DOI: 10.1097/mpg.0000000000003868.

35. t Hoen LA, Korfage IJ, Verhallen JT, et al. Vancouver Symptom Score for Dysfunctional Elimination Syndrome: Reliability and Validity of the Dutch Version. *J Urol* 2016; 196: 536-541.

36. Bower WF, Sit FKY, Bluyssen N, et al. PinQ: A valid, reliable and reproducible quality-of-life measure in children with bladder dysfunction. *Journal of Pediatric Urology* 2006; 2: 185-189. Article. DOI: 10.1016/j.jpurol.2005.07.004.

37. Lohuis PJFM, Hakim S, Duivesteijn W, et al. Benefits of a short, practical questionnaire to measure subjective perception of nasal appearance after aesthetic rhinoplasty. *Plastic and Reconstructive Surgery* 2013; 132: 913e-923e. Article. DOI: 10.1097/01.prs.0000434403.83692.95.

38. Klassen AF, Cano SJ, East CA, et al. Development and psychometric evaluation of the FACE-Q scales for patients undergoing rhinoplasty. *JAMA Facial Plastic Surgery* 2016; 18: 27-35. Article. DOI: 10.1001/jamafacial.2015.1445.

39. Moubayed SP, Ioannidis JPA, Saltychev M, et al. The 10-item Standardized Cosmesis and Health Nasal Outcomes Survey (SCHNOS) for functional and cosmetic rhinoplasty. *JAMA Facial Plastic Surgery* 2018; 20: 37-42. Article. DOI: 10.1001/jamafacial.2017.1083.

40. Tuinman MA, Gazendam-Donofrio SM and Hoekstra-Weebers JE. Screening and referral for psychosocial distress in oncologic practice: Use of the distress thermometer. *Cancer* 2008; 113: 870-878. Article. DOI: 10.1002/cncr.23622.

41. van Anrooij B, Kluin-Nelemans JC, Safy M, et al. Patient-reported disease-specific quality-of-life and symptom severity in systemic mastocytosis. *Allergy: European Journal of Allergy and Clinical Immunology* 2016; 71: 1585-1593. Article. DOI: 10.1111/all.12920.

42. Van Den Bergh RCN, Korfage IJ, Borsboom GJJM, et al. Prostate cancer-specific anxiety in Dutch patients on active surveillance: Validation of the memorial anxiety scale for prostate cancer. *Quality of Life Research* 2009; 18: 1061-1066. Article. DOI: 10.1007/s11136-009-9516-9.

43. Cocks K, Cohen D, Wisløff F, et al. An international field study of the reliability and validity of a disease-specific questionnaire module (the QLQ-MY20) in assessing the quality of life of patients with multiple myeloma. *European Journal of Cancer* 2007; 43: 1670-1678. Article. DOI: 10.1016/j.ejca.2007.04.022.

44. Veldhuijzen E, Walraven I, Mitchell SA, et al. Dutch translation and linguistic validation of the U.S. National Cancer Institute’s Patient-Reported Outcomes version of the Common Terminology Criteria for Adverse Events (PRO-CTCAE™). *Journal of Patient-Reported Outcomes* 2020; 4. Article. DOI: 10.1186/s41687-020-00249-y.

45. van der Meulen MJ, Lobbezoo F, John MT, et al. [Oral health impact profile. an instrument for measuring the impact of oral health on the quality of life]. *Nederlands tijdschrift voor tandheelkunde* 2011; 118: 134-139. Article. DOI: 10.5177/ntvt.2011.03.10178.

46. McGuire BE, Morrison TG, Hermanns N, et al. Short-form measures of diabetes-related emotional distress: The Problem Areas in Diabetes Scale (PAID)-5 and PAID-1. *Diabetologia* 2010; 53: 66-69. Article. DOI: 10.1007/s00125-009-1559-5.

47. Bastiaansen D, Koot HM, Bongers IL, et al. Measuring quality of life in children referred for psychiatric problems: Psychometric properties of the PedsQL™ 4.0 generic core scales. *Quality of Life Research* 2004; 13: 489-495. Article. DOI: 10.1023/B:QURE.0000018483.01526.ab.

48. De Zwart BCH, Frings-Dresen MHW and Van Duivenbooden JC. Test-retest reliability of the Work Ability Index questionnaire. *Occupational Medicine* 2002; 52: 177-181. Article. DOI: 10.1093/occmed/52.4.177.

49. Terwee CB, Roorda LD, De Vet HCW, et al. Dutch-Flemish translation of 17 item banks from the Patient-Reported Outcomes Measurement Information System (PROMIS). *Quality of Life Research* 2014; 23: 1733-1741. Article. DOI: 10.1007/s11136-013-0611-6.

50. Barentsen R, Van De Weijer PHM, Van Gend S, et al. Climacteric symptoms in a representative Dutch population sample as measured with the Greene Climacteric Scale. *Maturitas* 2001; 38: 123-128. Article. DOI: 10.1016/s0378-5122(00)00212-7.

51. Chan AHY, Horne R, Hankins M, et al. The Medication Adherence Report Scale: A measurement tool for eliciting patients' reports of nonadherence. *Br J Clin Pharmacol* 2020; 86: 1281-1288.

52. Bosscher RJ, Koning H and Van Meurs R. Reliability and validity of the Beck Depression Inventory in a Dutch college population. *Psychological reports* 1986; 58: 696-698. Article. DOI: 10.2466/pr0.1986.58.3.696.

53. Pellicciari L, Chiarotto A, Giusti E, et al. Psychometric properties of the patient-reported outcomes measurement information system scale v1.2: global health (PROMIS-GH) in a Dutch general population. *Health Qual Life Outcomes* 2021; 19: 226.

54. Luijten MAJ, Haverman L, van Litsenburg RRL, et al. Advances in measuring pediatric overall health: the PROMIS® Pediatric Global Health scale (PGH-7). *Eur J Pediatr* 2022; 181: 2117-2125.

55. Haverman L, Grootenhuis MA, Raat H, et al. Dutch–Flemish translation of nine pediatric item banks from the Patient-Reported Outcomes Measurement Information System (PROMIS)®. *Quality of Life Research* 2016; 25: 761-765. Article. DOI: 10.1007/s11136-015-0966-y.

56. Klaufus LH, Luijten MAJ, Verlinden E, et al. Psychometric properties of the Dutch-Flemish PROMIS® pediatric item banks Anxiety and Depressive Symptoms in a general population. *Quality of Life Research* 2021; 30: 2683-2695. Article. DOI: 10.1007/s11136-021-02852-y.

57. Gal R, van der Velden JM, Bach DC, et al. Translation and validation of the Dutch Spine Oncology Study Group Outcomes Questionnaire (SOSGOQ2.0) to evaluate health-related quality of life in patients with symptomatic spinal metastases. *BMC Musculoskeletal Disorders* 2022; 23. Article. DOI: 10.1186/s12891-022-05837-1.

58. Schlösser TPC, Stadhouder A, Schimmel JJP, et al. Reliability and validity of the adapted Dutch version of the revised Scoliosis Research Society 22-item questionnaire. *Spine Journal* 2014; 14: 1663-1672. Article. DOI: 10.1016/j.spinee.2013.09.046.

59. Santoso AMM, Lutomski JE, Hofman CS, et al. Development of a Patient-Reported Outcome Measure for Geriatric Care: The Older Persons and Informal Caregivers Survey Short Form. *Value in Health* 2018; 21: 1198-1204. Article. DOI: 10.1016/j.jval.2018.02.011.

60. Cleemput I and Dobbels F. Measuring patient-reported outcomes in solid organ transplant recipients: An overview of instruments developed to date. *PharmacoEconomics* 2007; 25: 269-286. Review. DOI: 10.2165/00019053-200725040-00002.

61. Badia X, Garcia-Losa M and Dal-Re R. Ten-language translation and harmonization of the international prostate symptom score: Developing a methodology for multinational clinical trials. *European Urology* 1997; 31: 129-140. Article. DOI: 10.1159/000474438.

62. Langelaan M, Van Nispen RMA, Knol DL, et al. Visual functioning questionnaire: Reevaluation of psychometric properties for a group of working-age adults. *Optometry and Vision Science* 2007; 84: 775-784. Article. DOI: 10.1097/OPX.0b013e3181334b98.

63. Pop VJ, Komproe IH and van Son MJ. Characteristics of the Edinburgh post natal depression scale in The Netherlands. *Journal of Affective Disorders* 1992; 26: 105-110. Article. DOI: 10.1016/0165-0327(92)90041-4.

64. Avery K, Donovan J, Peters TJ, et al. ICIQ: A brief and robust measure for evaluating the symptoms and impact of urinary incontinence. *Neurourology and Urodynamics* 2004; 23: 322-330. Review. DOI: 10.1002/nau.20041.

65. Jorge JMN and Wexner SD. Etiology and management of fecal incontinence. *Diseases of the Colon & Rectum* 1993; 36: 77-97. Article. DOI: 10.1007/bf02050307.

66. Emmens B, Hollins Martin CJ and Martin CR. Translation and validation of the Dutch version of the Birth Satisfaction Scale-Revised (BSS-R). *Journal of Reproductive and Infant Psychology* 2023; 41: 213-227. doi: 10.1080/02646838.2021.1979200. DOI: 10.1080/02646838.2021.1979200.

67. van Bussel JC, Spitz B and Demyttenaere K. Three self-report questionnaires of the early mother-to-infant bond: reliability and validity of the Dutch version of the MPAS, PBQ and MIBS. *Arch Womens Ment Health* 2010; 13: 373-384.

68. Dennis CL. The breastfeeding self-efficacy scale: psychometric assessment of the short form. *J Obstet Gynecol Neonatal Nurs* 2003; 32: 734-744.
